# 6-Bromo-4-oxo-4*H*-chromene-3-carb­alde­hyde

**DOI:** 10.1107/S160053681400796X

**Published:** 2014-04-16

**Authors:** Yoshinobu Ishikawa

**Affiliations:** a School of Pharmaceutical Sciences, University of Shizuoka, 52-1 Yada, Suruga-ku, Shizuoka 422-8526, Japan

## Abstract

In the title compound, C_10_H_5_BrO_3_, a brominated 3-formyl­chromone derivative, the non-H atoms are essentially coplanar (r.m.s. deviation = 0.0420 Å), with the largest deviation from its mean plane [0.109 (2) Å] being found for the ring-bound carbonyl O atom. In the crystal, mol­ecules are linked through halogen bonds [Br⋯O = 3.191 (2) Å, C—Br⋯O = 167.32 (10)° and C=O⋯Br = 168.4 (2)°] along [101]. Mol­ecules are assembled into layers parallel to (101) *via* π–π stacking inter­actions along the *b* axis [shortest centroid–centroid distance between the pyran and benzene rings = 3.495 (2) Å].

## Related literature   

For related structures, see: Ishikawa & Motohashi (2013 ▶); Ishikawa (2014*a* ▶,*b* ▶). For halogen bonding, see: Auffinger *et al.* (2004 ▶); Metrangolo *et al.* (2005 ▶); Wilcken *et al.* (2013 ▶); Sirimulla *et al.* (2013 ▶).
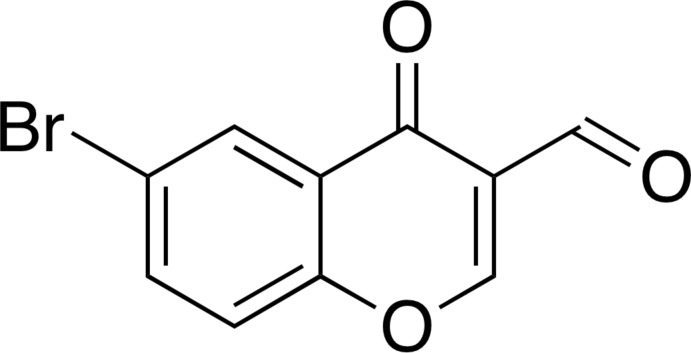


## Experimental   

### 

#### Crystal data  

C_10_H_5_BrO_3_
*M* *_r_* = 253.05
Triclinic, 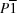

*a* = 6.5743 (18) Å
*b* = 6.967 (3) Å
*c* = 10.350 (4) Å
α = 71.02 (3)°
β = 85.53 (3)°
γ = 70.67 (3)°
*V* = 422.8 (3) Å^3^
*Z* = 2
Mo *K*α radiation
μ = 4.85 mm^−1^
*T* = 100 K
0.42 × 0.40 × 0.38 mm

#### Data collection  

Rigaku AFC-7R diffractometer
Absorption correction: ψ scan (North *et al.*, 1968 ▶) *T*_min_ = 0.135, *T*_max_ = 0.159
2389 measured reflections
1944 independent reflections
1880 reflections with *F* ^2^ > 2σ(*F* ^2^)
*R*_int_ = 0.024
3 standard reflections every 150 reflections intensity decay: 2.0%

#### Refinement  

*R*[*F* ^2^ > 2σ(*F* ^2^)] = 0.030
*wR*(*F* ^2^) = 0.077
*S* = 1.16
1944 reflections
128 parameters
H-atom parameters constrained
Δρ_max_ = 1.05 e Å^−3^
Δρ_min_ = −0.74 e Å^−3^

### 

Data collection: *WinAFC Diffractometer Control Software* (Rigaku, 1999 ▶); cell refinement: *WinAFC Diffractometer Control Software*; data reduction: *WinAFC Diffractometer Control Software*; program(s) used to solve structure: *SIR92* (Altomare, *et al.*, 1994 ▶); program(s) used to refine structure: *SHELXL97* (Sheldrick, 2008 ▶); molecular graphics: *CrystalStructure* (Rigaku, 2010 ▶); software used to prepare material for publication: *CrystalStructure*.

## Supplementary Material

Crystal structure: contains datablock(s) General, I. DOI: 10.1107/S160053681400796X/tk5306sup1.cif

Structure factors: contains datablock(s) I. DOI: 10.1107/S160053681400796X/tk5306Isup2.hkl

Supporting information file. DOI: 10.1107/S160053681400796X/tk5306Isup3.cml

CCDC reference: 996416

Additional supporting information:  crystallographic information; 3D view; checkCIF report

## Figures and Tables

**Fig. 1.**
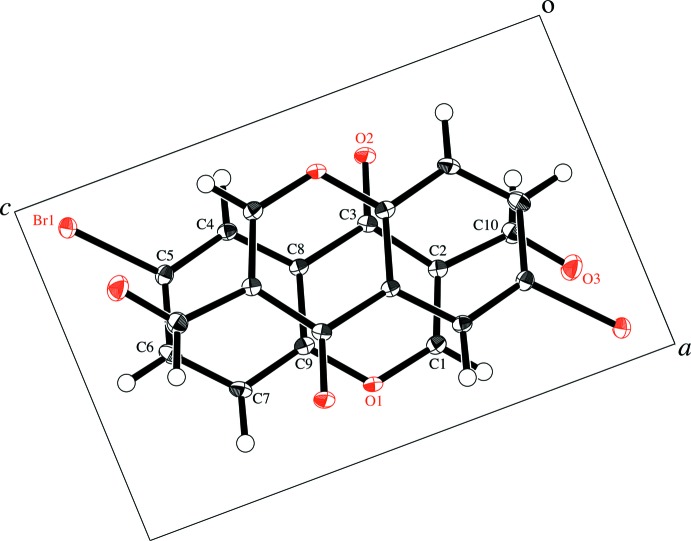
A packing view of the title compound with displacement ellipsoids drawn at the 50% probability level. Hydrogen atoms are shown as small spheres of arbitrary radius.

**Fig. 2.**
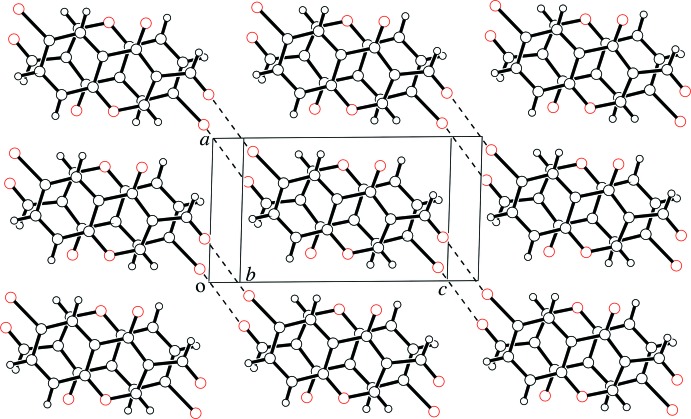
A packing view of the title compound. The intermolecular halogen bonds are represented as dashed lines for Br···O.

**Fig. 3.**
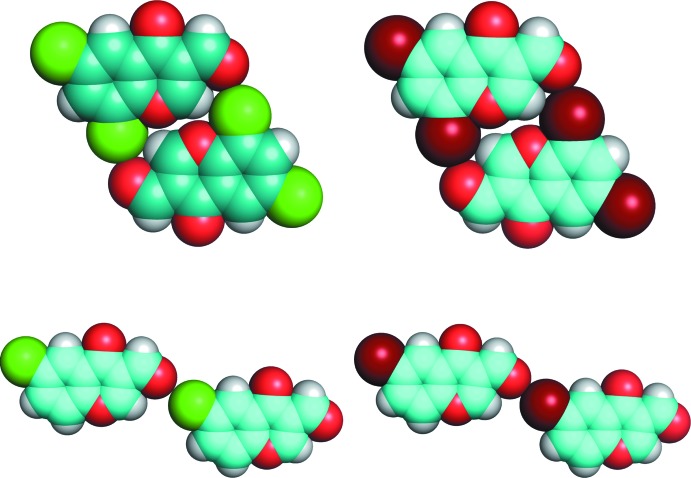
Sphere models of the crystal structures of 6,8-dichloro-4-oxo-4H-chromene-3-carbaldehyde (top left), 6,8-dibromo-4-oxo-4H-chromene-3-carbaldehyde (top right), 6-chloro-4-oxo-4H-chromene-3-carbaldehyde (bottom left), and the title compound (bottom right).

## supplementary crystallographic information

### 1. Structural commentary

Halogen bonds have been found to occur in organic, inorganic, and biological systems, and have recently attracted much attention in medicinal chemistry, chemical biology and supra­molecular chemistry (Auffinger *et al.*, 2004, Metrangolo *et al.*, 2005, Wilcken *et al.*, 2013, Sirimulla *et al.*, 2013). We have recently reported the crystal structures of dihalogenated 3-formyl­chromone derivatives 6,8-di­chloro-4-oxo-4*H*-chromene-3-carbaldehyde (Ishikawa & Motohashi, 2013, Fig.3 (top left)) and 6,8-di­bromo-4-oxo-4*H*-chromene-3-carbaldehyde (Ishikawa, 2014*a*, Fig.3 (top right)). It was found that halogen bonds between the formyl oxygen atom and the halogen atoms at 8-position are formed in those crystals in a similar fashion. On the other hand, halogen bond is not observed between any oxygen atom and the chlorine atom at 6-position in the crystal structure of 6-chloro-4-oxo-4*H*-chromene-3-carbaldehyde (Ishikawa, 2014*b*, Fig.3 (bottom left)). As part of our inter­est in this type of chemical bonding, we herein report the crystal structure of a monobrominated 3-formyl­chromone derivative 6-bromo-4-oxo-4*H*-chromene-3-carbaldehyde. The objective of this study is to reveal whether halogen bond(*s*) can be formed in the crystal structure of the title compound with the bromine atom at 6-position and without a halogen atom at 8-position.

The mean deviation of the least-square planes for the non-hydrogen atoms is 0.0420 Å, and the largest deviation is 0.109 (2) Å for O2. These mean that these atoms are essentially coplanar (Fig.1).

In the crystal, the molecules are stacked with the inversion-symmetry equivalent^i^ along the *b*-axis direction [shortest centroid–centroid distance between the pyran and benzene rings of the 4*H*-chromene units = 3.495 (2) Å, i: -*x* + 1, -*y* + 1, -*z* + 1], as shown in Fig. 1. The *Cg*–*Cg* distance of the title compound is almost equal to that of 6-chloro-4-oxo-4*H*-chromene-3-carbaldehyde (3.4959 (15) Å, Ishikawa, 2014*b*).

Halogen bond was observed between the bromine atom at 6-position and the formyl oxygen atom of the translation-symmetry equivalent^ii^ [Br1···O3^ii^ = 3.191 (2) Å, ii: *x* - 1, *y*, *z* + 1] along [101], as shown in Fig.2. The angles of C–Br···O and Br···O=C are 167.32 (10) and 168.4 (2)°, respectively. Thus, it is found that halogen bonds are formed for the bromine atoms not only at 8-position but also at 6-position, as shown in the top right and bottom right of Fig.3.

The space group and crystal packing mode of the title compound are the same with those of 6-chloro-4-oxo-4*H*-chromene-3-carbaldehyde. On the other hand, halogen bond is observed in the former and not in the latter, as shown in the bottom of Fig.3. These should be accounted for by the larger size of the σ hole of the bromine atom at 6-position (Wilcken *et al.*, 2013). These results might be applicable for rational drug design.

### 2. Synthesis and crystallization

Single crystals suitable for X-ray diffraction were obtained by slow evaporation of an *N*,*N*-di­methyl­formamide solution of the commercially available title compound at room temperature.

### 3. Refinement

Crystal data, data collection and structure refinement details are summarized in Table 1. The C(*sp*^2^)-bound hydrogen atoms were placed in geometrical positions [C–H 0.95 Å, *U*_iso_(H) = 1.2*U*_eq_(C)], and refined using a riding model.

### Crystal data

**Table:**

C_10_H_5_BrO_3_	*Z* = 2
*M**_r_* = 253.05	*F*(000) = 248.00
Triclinic, *P*1	*D*_x_ = 1.988 Mg m^−^^3^
Hall symbol: -P 1	Mo *K*α radiation, λ = 0.71069 Å
*a* = 6.5743 (18) Å	Cell parameters from 25 reflections
*b* = 6.967 (3) Å	θ = 15.2–17.4°
*c* = 10.350 (4) Å	µ = 4.85 mm^−^^1^
α = 71.02 (3)°	*T* = 100 K
β = 85.53 (3)°	Block, colorless
γ = 70.67 (3)°	0.42 × 0.40 × 0.38 mm
*V* = 422.8 (3) Å^3^	

### Data collection

**Table:**

Rigaku AFC-7R diffractometer	*R*_int_ = 0.024
ω–2θ scans	θ_max_ = 27.5°
Absorption correction: ψ scan (North *et al.*, 1968)	*h* = −8→8
*T*_min_ = 0.135, *T*_max_ = 0.159	*k* = −5→9
2389 measured reflections	*l* = −12→13
1944 independent reflections	3 standard reflections every 150 reflections
1880 reflections with *F*^2^ > 2σ(*F*^2^)	intensity decay: 2.0%

### Refinement

**Table:**

Refinement on *F*^2^	Hydrogen site location: inferred from neighbouring sites
*R*[*F*^2^ > 2σ(*F*^2^)] = 0.030	H-atom parameters constrained
*wR*(*F*^2^) = 0.077	*w* = 1/[σ^2^(*F*_o_^2^) + (0.0545*P*)^2^ + 0.1959*P*] where *P* = (*F*_o_^2^ + 2*F*_c_^2^)/3
*S* = 1.16	(Δ/σ)_max_ = 0.001
1944 reflections	Δρ_max_ = 1.05 e Å^−^^3^
128 parameters	Δρ_min_ = −0.74 e Å^−^^3^
0 restraints	Extinction correction: *SHELXL97* (Sheldrick, 2008)
Primary atom site location: structure-invariant direct methods	Extinction coefficient: 0.151 (9)
Secondary atom site location: difference Fourier map	

### Special details

**Table:**

Refinement. Refinement was performed using all reflections. The weighted *R*-factor (*wR*) and goodness of fit (*S*) are based on *F*^2^. *R*-factor (gt) are based on *F*. The threshold expression of *F*^2^ > 2.0 σ(*F*^2^) is used only for calculating *R*-factor (gt).

### Fractional atomic coordinates and isotropic or equivalent isotropic displacement parameters (Å^2^)

**Table:**

	*x*	*y*	*z*	*U*_iso_*/*U*_eq_	
Br1	0.09454 (3)	0.28528 (3)	0.922893 (19)	0.01450 (13)	
O1	0.8085 (3)	0.1862 (3)	0.52565 (16)	0.0122 (3)	
O2	0.1989 (3)	0.3151 (3)	0.38347 (17)	0.0157 (4)	
O3	0.6969 (3)	0.2664 (3)	0.11860 (18)	0.0203 (4)	
C1	0.7668 (4)	0.2190 (4)	0.3943 (3)	0.0120 (4)	
C2	0.5687 (4)	0.2640 (3)	0.3402 (2)	0.0101 (4)	
C3	0.3797 (4)	0.2830 (3)	0.4253 (2)	0.0100 (4)	
C4	0.2621 (4)	0.2805 (3)	0.6624 (2)	0.0106 (4)	
C5	0.3145 (4)	0.2506 (4)	0.7956 (3)	0.0112 (4)	
C6	0.5287 (4)	0.1952 (4)	0.8394 (3)	0.0136 (4)	
C7	0.6918 (4)	0.1709 (4)	0.7478 (3)	0.0132 (4)	
C8	0.4269 (3)	0.2578 (4)	0.5684 (2)	0.0092 (4)	
C9	0.6393 (4)	0.2048 (3)	0.6128 (3)	0.0107 (4)	
C10	0.5462 (4)	0.2943 (4)	0.1932 (3)	0.0145 (4)	
H1	0.8846	0.2100	0.3347	0.0143*	
H2	0.1162	0.3159	0.6347	0.0127*	
H3	0.5614	0.1744	0.9318	0.0163*	
H4	0.8378	0.1315	0.7765	0.0158*	
H5	0.4050	0.3382	0.1550	0.0174*	

### Atomic displacement parameters (Å^2^)

**Table:**

	*U*^11^	*U*^22^	*U*^33^	*U*^12^	*U*^13^	*U*^23^
Br1	0.01323 (17)	0.01865 (17)	0.01105 (16)	−0.00255 (10)	0.00148 (9)	−0.00690 (10)
O1	0.0070 (7)	0.0168 (8)	0.0124 (8)	−0.0032 (6)	−0.0008 (6)	−0.0044 (6)
O2	0.0099 (7)	0.0254 (9)	0.0140 (8)	−0.0066 (6)	−0.0013 (6)	−0.0079 (7)
O3	0.0184 (8)	0.0302 (10)	0.0172 (9)	−0.0093 (7)	0.0048 (7)	−0.0133 (8)
C1	0.0107 (9)	0.0122 (9)	0.0133 (10)	−0.0034 (8)	0.0011 (8)	−0.0050 (8)
C2	0.0104 (9)	0.0089 (9)	0.0120 (10)	−0.0032 (7)	−0.0000 (8)	−0.0045 (7)
C3	0.0103 (9)	0.0085 (9)	0.0118 (10)	−0.0032 (7)	−0.0012 (8)	−0.0037 (7)
C4	0.0095 (9)	0.0104 (9)	0.0126 (10)	−0.0032 (7)	−0.0001 (8)	−0.0047 (8)
C5	0.0122 (9)	0.0110 (9)	0.0116 (10)	−0.0040 (7)	0.0019 (8)	−0.0055 (7)
C6	0.0154 (10)	0.0122 (10)	0.0139 (10)	−0.0045 (8)	−0.0037 (8)	−0.0043 (8)
C7	0.0107 (9)	0.0144 (10)	0.0143 (11)	−0.0041 (8)	−0.0038 (8)	−0.0035 (8)
C8	0.0087 (9)	0.0085 (8)	0.0117 (10)	−0.0035 (7)	0.0001 (8)	−0.0043 (7)
C9	0.0095 (9)	0.0088 (9)	0.0135 (10)	−0.0032 (7)	0.0004 (8)	−0.0032 (7)
C10	0.0153 (10)	0.0174 (10)	0.0139 (10)	−0.0069 (8)	0.0005 (8)	−0.0075 (8)

### Geometric parameters (Å, º)

**Table:**

Br1—C5	1.888 (3)	C4—C8	1.403 (3)
O1—C1	1.338 (3)	C5—C6	1.401 (4)
O1—C9	1.375 (3)	C6—C7	1.380 (4)
O2—C3	1.221 (3)	C7—C9	1.391 (4)
O3—C10	1.211 (3)	C8—C9	1.395 (3)
C1—C2	1.356 (4)	C1—H1	0.950
C2—C3	1.461 (3)	C4—H2	0.950
C2—C10	1.478 (4)	C6—H3	0.950
C3—C8	1.480 (4)	C7—H4	0.950
C4—C5	1.379 (4)	C10—H5	0.950
			
O1···C3	2.868 (3)	C9···H1	3.1828
O2···C1	3.572 (3)	C9···H2	3.2719
O2···C4	2.869 (3)	C9···H3	3.2502
O2···C10	2.894 (3)	C10···H1	2.5537
O3···C1	2.820 (4)	H1···H5	3.4881
C1···C7	3.574 (4)	H3···H4	2.3392
C1···C8	2.757 (3)	Br1···H3^xi^	3.1998
C2···C9	2.768 (4)	Br1···H4^vi^	2.9904
C4···C7	2.805 (4)	Br1···H4^xi^	3.4343
C5···C9	2.748 (4)	Br1···H5^x^	3.4515
C6···C8	2.795 (4)	Br1···H5^vii^	3.4131
Br1···O3^i^	3.191 (2)	O1···H1^ii^	2.8201
O1···O1^ii^	3.117 (3)	O1···H2^iii^	2.9005
O1···O2^iii^	3.104 (3)	O1···H2^v^	3.5064
O1···O2^iv^	3.325 (3)	O2···H1^vi^	2.5430
O1···C1^ii^	3.174 (3)	O2···H2^vii^	2.6756
O1···C4^v^	3.479 (3)	O3···H3^ix^	2.5290
O1···C8^v^	3.488 (3)	O3···H3^v^	3.5734
O2···O1^vi^	3.104 (3)	O3···H4^ii^	3.3419
O2···O1^iv^	3.325 (3)	O3···H5^xii^	3.1712
O2···C1^vi^	3.113 (4)	C1···H2^v^	3.4876
O2···C4^vii^	3.325 (3)	C3···H2^vii^	3.4612
O2···C9^iv^	3.408 (4)	C4···H1^v^	3.3664
O3···Br1^viii^	3.191 (2)	C4···H4^vi^	3.2949
O3···C5^iv^	3.444 (4)	C5···H1^v^	3.3731
O3···C6^ix^	3.408 (4)	C5···H3^xi^	3.2745
C1···O1^ii^	3.174 (3)	C5···H4^vi^	3.5319
C1···O2^iii^	3.113 (4)	C6···H3^xi^	3.0684
C1···C4^v^	3.285 (4)	C6···H5^iv^	3.5775
C1···C5^v^	3.455 (4)	C6···H5^v^	3.4388
C1···C8^v^	3.581 (4)	C7···H1^ii^	3.4259
C2···C4^iv^	3.591 (4)	C7···H2^iii^	3.2813
C2···C5^v^	3.530 (4)	C9···H1^ii^	3.4206
C2···C6^v^	3.478 (4)	C9···H2^iii^	3.5095
C2···C7^v^	3.566 (4)	C10···H3^ix^	3.0662
C2···C8^iv^	3.437 (4)	C10···H3^v^	3.3423
C3···C3^iv^	3.568 (3)	H1···O1^ii^	2.8201
C3···C7^v^	3.525 (4)	H1···O2^iii^	2.5430
C3···C8^iv^	3.535 (4)	H1···C4^v^	3.3664
C3···C9^iv^	3.588 (4)	H1···C5^v^	3.3731
C3···C9^v^	3.422 (4)	H1···C7^ii^	3.4259
C4···O1^v^	3.479 (3)	H1···C9^ii^	3.4206
C4···O2^vii^	3.325 (3)	H1···H2^iv^	3.5756
C4···C1^v^	3.285 (4)	H1···H2^v^	3.4176
C4···C2^iv^	3.591 (4)	H1···H4^ii^	2.9827
C4···C10^iv^	3.594 (4)	H2···O1^vi^	2.9005
C5···O3^iv^	3.444 (4)	H2···O1^v^	3.5064
C5···C1^v^	3.455 (4)	H2···O2^vii^	2.6756
C5···C2^v^	3.530 (4)	H2···C1^v^	3.4876
C5···C10^iv^	3.563 (4)	H2···C3^vii^	3.4612
C6···O3^x^	3.408 (4)	H2···C7^vi^	3.2813
C6···C2^v^	3.478 (4)	H2···C9^vi^	3.5095
C6···C10^v^	3.331 (4)	H2···H1^iv^	3.5756
C7···C2^v^	3.566 (4)	H2···H1^v^	3.4176
C7···C3^v^	3.525 (4)	H2···H2^vii^	3.1789
C8···O1^v^	3.488 (3)	H2···H4^vi^	2.6584
C8···C1^v^	3.581 (4)	H3···Br1^xi^	3.1998
C8···C2^iv^	3.437 (4)	H3···O3^x^	2.5290
C8···C3^iv^	3.535 (4)	H3···O3^v^	3.5734
C8···C9^v^	3.494 (4)	H3···C5^xi^	3.2745
C9···O2^iv^	3.408 (4)	H3···C6^xi^	3.0684
C9···C3^iv^	3.588 (4)	H3···C10^x^	3.0662
C9···C3^v^	3.422 (4)	H3···C10^v^	3.3423
C9···C8^v^	3.494 (4)	H3···H3^xi^	2.7283
C10···C4^iv^	3.594 (4)	H3···H5^x^	2.8751
C10···C5^iv^	3.563 (4)	H3···H5^v^	3.2964
C10···C6^v^	3.331 (4)	H4···Br1^iii^	2.9904
Br1···H2	2.9161	H4···Br1^xi^	3.4343
Br1···H3	2.9076	H4···O3^ii^	3.3419
O1···H4	2.5120	H4···C4^iii^	3.2949
O2···H2	2.6160	H4···C5^iii^	3.5319
O2···H5	2.6169	H4···H1^ii^	2.9827
O3···H1	2.4933	H4···H2^iii^	2.6584
C1···H5	3.2796	H5···Br1^ix^	3.4515
C3···H1	3.2958	H5···Br1^vii^	3.4131
C3···H2	2.6880	H5···O3^xii^	3.1712
C3···H5	2.6959	H5···C6^iv^	3.5775
C4···H3	3.2780	H5···C6^v^	3.4388
C5···H4	3.2671	H5···H3^ix^	2.8751
C6···H2	3.2828	H5···H3^v^	3.2964
C8···H4	3.2883		
			
C1—O1—C9	118.51 (18)	C4—C8—C9	118.9 (2)
O1—C1—C2	124.7 (2)	O1—C9—C7	116.04 (19)
C1—C2—C3	120.7 (2)	O1—C9—C8	122.2 (2)
C1—C2—C10	119.0 (2)	C7—C9—C8	121.7 (2)
C3—C2—C10	120.3 (2)	O3—C10—C2	124.0 (3)
O2—C3—C2	123.6 (3)	O1—C1—H1	117.637
O2—C3—C8	122.6 (2)	C2—C1—H1	117.636
C2—C3—C8	113.81 (19)	C5—C4—H2	120.442
C5—C4—C8	119.1 (2)	C8—C4—H2	120.436
Br1—C5—C4	119.78 (16)	C5—C6—H3	120.165
Br1—C5—C6	118.71 (18)	C7—C6—H3	120.161
C4—C5—C6	121.5 (2)	C6—C7—H4	120.488
C5—C6—C7	119.7 (3)	C9—C7—H4	120.487
C6—C7—C9	119.0 (2)	O3—C10—H5	118.015
C3—C8—C4	121.2 (2)	C2—C10—H5	118.010
C3—C8—C9	119.87 (19)		
			
C1—O1—C9—C7	−179.47 (17)	C8—C4—C5—Br1	178.91 (17)
C1—O1—C9—C8	−0.5 (3)	C8—C4—C5—C6	−1.0 (4)
C9—O1—C1—C2	−1.9 (3)	H2—C4—C5—Br1	−1.1
C9—O1—C1—H1	178.1	H2—C4—C5—C6	179.0
O1—C1—C2—C3	0.8 (4)	H2—C4—C8—C3	−1.5
O1—C1—C2—C10	−179.35 (18)	H2—C4—C8—C9	−179.7
H1—C1—C2—C3	−179.2	Br1—C5—C6—C7	−179.45 (14)
H1—C1—C2—C10	0.6	Br1—C5—C6—H3	0.6
C1—C2—C3—O2	−177.1 (2)	C4—C5—C6—C7	0.4 (4)
C1—C2—C3—C8	2.4 (3)	C4—C5—C6—H3	−179.6
C1—C2—C10—O3	5.5 (4)	C5—C6—C7—C9	0.8 (4)
C1—C2—C10—H5	−174.5	C5—C6—C7—H4	−179.2
C3—C2—C10—O3	−174.6 (2)	H3—C6—C7—C9	−179.2
C3—C2—C10—H5	5.4	H3—C6—C7—H4	0.8
C10—C2—C3—O2	3.0 (4)	C6—C7—C9—O1	177.51 (19)
C10—C2—C3—C8	−177.47 (18)	C6—C7—C9—C8	−1.5 (4)
O2—C3—C8—C4	−3.2 (4)	H4—C7—C9—O1	−2.5
O2—C3—C8—C9	174.99 (19)	H4—C7—C9—C8	178.5
C2—C3—C8—C4	177.26 (17)	C3—C8—C9—O1	3.8 (4)
C2—C3—C8—C9	−4.5 (3)	C3—C8—C9—C7	−177.29 (18)
C5—C4—C8—C3	178.50 (18)	C4—C8—C9—O1	−177.99 (18)
C5—C4—C8—C9	0.3 (3)	C4—C8—C9—C7	1.0 (4)

Symmetry codes: (i) *x*−1, *y*, *z*+1; (ii) −*x*+2, −*y*, −*z*+1; (iii) *x*+1, *y*, *z*; (iv) −*x*+1, −*y*, −*z*+1; (v) −*x*+1, −*y*+1, −*z*+1; (vi) *x*−1, *y*, *z*; (vii) −*x*, −*y*+1, −*z*+1; (viii) *x*+1, *y*, *z*−1; (ix) *x*, *y*, *z*−1; (x) *x*, *y*, *z*+1; (xi) −*x*+1, −*y*, −*z*+2; (xii) −*x*+1, −*y*+1, −*z*.
